# Interventions to Reduce and Prevent Obesity in Pre-Conceptual and Pregnant Women: A Systematic Review and Meta-Analysis

**DOI:** 10.1371/journal.pone.0095132

**Published:** 2014-05-14

**Authors:** Maliha Agha, Riaz A. Agha, Jane Sandell

**Affiliations:** 1 King's College London, London, United Kingdom; 2 Queen Victoria Hospital NHS Foundation Trust, East Grinstead, United Kingdom; 3 Women's Academic Health Centre, King's College London, London, United Kingdom; University of Missouri, United States of America

## Abstract

**Background:**

The increasing prevalence of obesity in pregnant women is associated with adverse maternal and neonatal outcomes, and increased costs to healthcare, the economy and broader society.

**Objectives:**

To assess the efficacy of behavioural interventions for managing gestational weight gain (GWG) in the pre-conceptual and pregnancy period in overweight, obese and morbidly obese women.

**Search Methods:**

A search was performed for published studies in the English language, from date? 2000–31 December 2012 in five electronic databases; PubMed, Scopus, Cochrane Library, CINAHL and PsycINFO.

**Selection criteria:**

Studies were included if they compared the efficacy or effectiveness of a particular behavioural intervention in pregnant or pre-conceptual women with standard maternity care. Studies that included women with co-morbid conditions such as diabetes mellitus and polycystic ovarian syndrome were excluded to help isolate the effect of the intervention.

**Results:**

Fifteen studies involving 3,426 participants were included. One study (n = 692) focused on the pre-conceptual period and the remaining 14 (n = 2,734) in the pregnancy period. Pooled mean difference for GWG indicated a lower GWG in the intervention groups when compared to standard maternity care groups (n = 1771, mean difference (MD) −1.66 kg, 95% CI −3.12 to −0.21 kg). With respect to the types of participants, considerable heterogeneity between studies was shown in the obese subgroup [Tau^2^ = 15.61; Chi^2^ = 40.80, df = 3 (P<0.00001); I^2^ = 93%].

**Conclusions:**

Behavioural interventions in pregnancy may be effective in reducing GWG in obese women without comorbid conditions, but not overweight or morbidly obese women. Behavioural interventions had no effect on postpartum weight loss or retention, gestation week of delivery and infant birth weight in overweight, obese and morbidly obese women.

## Introduction

Overweight (Body mass index or BMI ≥25 kg/m^2^) and obesity (BMI ≥30 kg/m^2^) is a risk factor for; cardiovascular diseases (CVD), diabetes, musculoskeletal disorders and certain cancers [1]–[2]. The World Health Organisation (WHO) has described overweight and obesity as the fifth most important risk factor for global deaths [3]. In 2008, nearly 300 million women and 200 million men were obese [2]. By 2015, this is expected to rise 40% to 700 million [4].

Gestational weight gain (GWG) is an important metric to consider when discussing obesity before, during and after pregnancy. Less GWG is associated with better maternal outcomes [5]. Increased GWG is associated with improvements in some infant health outcomes, such as full-term birth and infant mortality, but greater risk for others, including increased foetal growth [6]–[7]. Therefore, debate has existed over the optimal range of GWG for both mothers and their babies.

Studies have shown a significant correlation between the pre-pregnancy weight and GWG [3], [8], [9]. Goals of a successful pregnancy weight gain should be established by women planning to get pregnant on the basis of their pre-pregnancy BMI [10], [11]. GWG appears to increase maternal fat stores rather than birth weight in obese women [12], [13], [14].

The maternal health risks of gestational obesity during pregnancy include; backaches, leg pain, increased fatigue, gestational diabetes (OR 3.6, 95% CI 3.3–4.0) [15], pre-eclampsia (OR 3.3, 95% CI 2.7–3.9) [16], thromboembolism (OR 9.7, 95% CI 3.1–30.8) [17], slow labour progress (p<0.001) [18], high caesarean section rates, postpartum haemorrhage (OR 1.4, 95% CI 1.2–1.6) [15], maternal death [19] and hypertension [15].

Foetal risks associated with maternal obesity during pregnancy include; miscarriage, foetal congenital anomaly, macrosomia [20], shoulder dystocia, stillbirth (OR 2.1, 95% CI 1.5–2.7) [21] and neonatal death (OR 2.6, 95% CI 1.2–5.8) [22]. Other post delivery complications include; low breast-feeding rates [23]–[24], caesarean wound infection (OR 2.24, 95% CI 1.91–2.64) [15] and postpartum weight retention [25]–[26].

Almost one in five pregnant women is obese in the UK today [27]. The prevalence of obesity in pregnancy is a major public health problem and has been rising since 1993, in the UK [27]. Around 50% of women of childbearing age were overweight or obese in 2006 [28]. According to the Health Survey for England (HSE), women of child bearing age starting pregnancy in an obese condition have increased from 12% in 1993 to 18.5% in 2006 [29]. Due to its magnitude and impact on women's reproductive health and their babies, obesity is one of the biggest challenges faced by the maternity services in the UK today [30].

There are no clinical guidelines on the most effective intervention for weight management in pre-conceptual and pregnant women and the paucity of appropriately and efficaciously designed interventions for maternal obesity is the basis for our investigation [31].

## Objectives

### Primary Objectives

1. To assess the efficacy of behavioural interventions for weight-management during pregnancy in overweight, obese and morbidly obese women.
2. In pre-conceptual and pregnant women, what is the effect of a particular behavioural intervention on excessive GWG compared with standard maternity care or no care, and what is the methodological quality of the evidence?

Excessive GWG was defined as GWG beyond that normally expected as part of the pregnancy as defined in Institute of Medicine (IOM) guidelines. The extra weight gained normally expected by women during pregnancy is derived from a variety of sources including the baby, placenta, amniotic fluid, uterus, maternal blood, fluids in maternal tissue and maternal fat and nutrient stores [32]. Weight gain during pregnancy should depend (at least in part) on the mother's weight before pregnancy and the stage of pregnancy. Studies have shown a significant correlation between the pre-pregnancy weight and GWG [3], [33]–[34]. Goals of a successful pregnancy weight gain should be established by women planning to get pregnant on the basis of their pre-pregnancy BMI [1]–[2], [35]–[36].

### Secondary Objectives

1. To evaluate which stage (pre-conceptual or pregnancy) is the most effective stage to affect the outcomes.
2. To assess the efficacy of interventions for pre-conceptual and pregnant women in producing an impact on postpartum weight loss, postpartum weight retention, gestation week of delivery and infant birth weight.
3. To perform subgroup and sensitivity analysis of the primary outcome (GWG) in the presence of substantial heterogeneity.

## Method

### Criteria for Considering Studies for this Review

#### Types of studies

The study had to be written in the English language and be a parallel group study evaluating the efficacy of behavioural interventions on weight management with standard maternity care or no intervention/placebo were included, for women planning to get pregnant or those who are already pregnant. Studies were eligible if at least one review outcome measure was reported. Based on criteria established by the Cochrane Effective Practice and Organisation of Care Group (EPOC), four types of study designs were eligible:

1. Randomised Controlled Trials (RCT).
2. Controlled Clinical Trials (CCT).
3. Controlled Before and After Studies (CBA).
4. Interrupted Time Series Design (ITS).

#### Types of participants

To be included, studies were required to include women of child-bearing age planning to get pregnant and/or those who were already pregnant. Studies including women who were classified as; underweight, normal weight, overweight, obese or morbidly obese were included. No limitations in relation to age, ethnicity, socio-economic status or body weight at enrolment were set. We excluded studies of women with conditions such as diabetes mellitus (DM) and polycystic ovary syndrome (POS) as these could represent potential confounders for observed differences.

#### Types of interventions

Interventions based on weight-reducing pharmaceutical or surgical interventions were not included. Interventions aimed at other conditions or diseases during pregnancy such as POS and DM and those purely aimed at postpartum weight management were excluded.

#### Types of outcome measures

The primary outcome was total GWG. Secondary outcomes were postpartum weight retention, postpartum weight loss, infant birth weight and gestation week of delivery.

### Search Terms and Keywords

The search terms used across all the databases were:

“Pregnancy” AND “Obesity” AND “Intervention”.

#### Electronic searches

A comprehensive computerised literature search of published studies was identified by searching the electronic databases; Pub Med, Scopus, Cochrane Library, CINAHL and PsycINFO. No limitations were applied on the basis of country and type of study. The last literature search was concluded on 31 December 2012. All searches were conducted by MA (and double checked by JS to minimise human error).

#### Searching other resources

The reference list and bibliography of all the studies selected during the abstract screening stage was also reviewed. Other strategies used included scanning the reference list and bibliography of existing NICE guidance on obesity, this search was concluded on 12 May 2011. The clinical Trials Registry of the US National Institutes of Health and Google Scholar were also searched.

The Office for National Statistics (ONS) [37] and National Obesity Observatory (NOO) [38] were contacted for statistics on obesity in the UK. Personal communication was made with NICE for further information and additional perspectives on ongoing systematic reviews.

### Data Collection and Analysis

#### Selection of studies

All potentially relevant articles were reviewed separately by the first reviewer, MA, and final selections were based on consensus reached through discussions with the second reviewer JS. Screening occurred in two stages. First the titles and abstracts were screened to identify any potentially relevant studies. Second, the full text of potentially relevant studies were reviewed to determine whether they met all eligibility criteria.

#### Study Validity and Methodological Quality

The **validity** of study design and methodological details was assessed using Cochrane validity criteria [3] as a starting point. Two groups were created at this stage; studies assessing pre-conceptual interventions were placed in one group (PC) and studies assessing pregnancy interventions were placed in another (DP). A unique ID was assigned to studies with multiple publications for the purpose of convenience and all outputs from each study were collated. This was also helpful to avoid data duplication when performing meta-analysis.

Primary validity assessment of study design was as follows;

- “Done” if the study design was relevant to EPOC study design inclusion criteria.
- “Not done” if study design was irrelevant to EPOC study design inclusion criteria.
- “Not clear” if study design was not clearly stated.

Secondary validity assessment of methodological details was performed on the basis of either outcomes that were reported qualitatively or quantitatively and if data presented was clear. It was scored as follows:

- “Done” if data was presentable and obtainable and outcomes are measured.
- “Not done” if relevant data was not presented; if data was self-reported and not measured.
- “Not clear” if data presented is not clear and requires contact with the author of the paper for clarification before data extraction.

Two reviewers reviewed the full text of all potentially relevant studies for relevance independently, in a non-blinded standardised manner with authors and institutions visible to the assessor. Any disagreements between the reviewers were resolved through discussion.

### Data Extraction and Management

Data extraction was completed by one reviewer (MA) using a pre-defined form, then checked for accuracy by a second reviewer (JS). There were no discrepancies between reviewers in terms of data extracted or choice of articles meriting inclusion and one hundred percent agreement between the reviewers was observed at all stages. The data extraction form included 47 items of data including; participant characteristics e.g., age, pre-pregnancy BMI, gestational age at recruitment, parity, socioeconomic status - employment and education, and other health behaviours such as smoking, study design, time period and location of research and primary purpose of the study, intervention characteristics including type, contents and mode of delivery of interventions, who delivered the intervention, setting where interventions were delivered, beginning of intervention and either the intervention was delivered in a group or to individual participants. Further details on the type and nature of the controls were also collected for comparison.

Data was also collected on inclusion and exclusion criteria of participants in the studies. Further details on the assessment and number of participants included in the study and the number of participants who completed the study at each assessment stage was also determined. From this data attrition (loss to follow-up) was calculated.

#### Outcomes

All the primary and secondary outcomes determined by each trial was recorded and presented in this section.

### Assessment of Methodological Quality of Included Studies

One reviewer (MA) independently assessed the risk of bias of included studies using the Cochrane Risk of Bias tool. The specific domains included; random sequence generation, allocation concealment, blinding, incomplete outcome data and selective reporting. Quality assessment was double checked by the senior author (JS). Any disagreement was resolved by discussion between the two reviewers. The judgements are described qualitatively in the table of assessment of bias.

Once the risk of bias was qualitatively assessed and recorded, the assessment result was further quantified using a scoring system.

- A score of 2 was allocated for judgements that were “Adequate”.
- A score of 1 was allocated for judgements that were “Unclear”.
- A score of 0 was allocated for judgements that were “Inadequate”.
- A score of 2 was allocated for judgements that were “Yes”.
- A score of 0 was allocated for judgements that were “No”.

### Ethical approval

As this was a systematic review of literature, ethics committee approval was not required.

## Meta-Analysis – Method

There is no accepted standardised approach to assess and measure gestational weight gain (GWG), in seven studies [DP1, DP6, DP8, DP10, DP11, DP14, DP19], GWG was calculated based on pre-pregnancy weight, in two studies [DP7, DP9] it was based on weight during early pregnancy. Therefore, the results for GWG were summarized using a standardized mean difference with 95% confidence intervals. The results for the following outcomes were summarized using a mean difference with 95% confidence intervals: postpartum weight loss (PWL), postpartum weight retention (PWR) (all in kg), gestation week of delivery (GWD), and infant birth weight (in g). Where necessary, outcomes were converted from pounds (lb) to kilogram (kg) by using the formula 1lb = 0.45359237 kg and kilogram was converted to gram (g) by using the formula 1kg  = 1000 g. Studies reporting gestation days of delivery were converted to weeks by dividing the days by 7. Where appropriate, meta-analysis was carried out using Review Manager 5.1 (RevMan).

### Assessment of Heterogeneity

Potential sources of heterogeneity were explored by performing subgroup analysis based on the following variables: pre-pregnancy BMI of the participants included in the studies (all weight versus participants classified as obese), types of intervention (passive versus pro-active interventions), psychological theory used in interventions (regular feedback and/or weight monitoring versus no feedback and/or weight monitoring), whether the quality of the studies (based on total score) was high or low, and place of 'delivery of intervention (community versus hospital based setting). Subgroup analyses were restricted to the review's primary outcome only (GWG).

Variability in the intervention effects for each outcome in studies was tested by statistical heterogeneity, using the Tau-squared (T^2^), I^2^, and chi-squared (Chi^2^) statistics with its corresponding P-value (the Cochrane tests). The I^2^ percentage was interpreted as follows:

- 0%–40% may not be important.
- 30%–60% may represent moderate heterogeneity*
- 50%–90% may represent substantial heterogeneity*
- 75%–100% represents considerable heterogeneity*

Publication bias for each outcome was assessed by using a funnel plot.

### Sensitivity Analysis

A sensitivity analysis in addition to subgroup analysis on primary outcome (GWG) was used to explore substantial heterogeneity. The sensitivity analysis decision was made by using decision nodes stated in the Cochrane Handbook of systematic reviews [39]. Since there is no accepted standardised approach to assess and measure GWG; in seven studies (DP1, DP6, DP8, DP10, DP11, DP14 and DP19) GWG was calculated based on pre-pregnancy weight, in two studies (DP7 and DP9) it was based on weight during early pregnancy and in the other two studies (DP12 and DP18) it was not reported. Therefore, despite the fact that GWG in early pregnancy may usually be low, to reduce imprecision in measurement, a correct measurement strategy of GWG to quantify the effect of the intervention could be established. Therefore, the results were assessed again by using standardised mean difference (SMD) across studies included in GWG.

The differences in heterogeneity obtained by using mean difference and standardised mean difference were compared and interpreted. The use of SMD is a standard procedure implemented in the Review Manager software.

## Results

### Description of Studies

**Figure 1:**
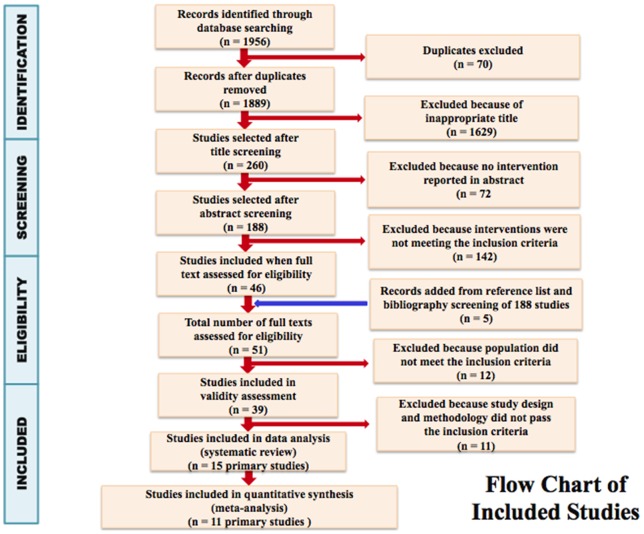
PRISMA flow diagram.

Search of the electronic databases elicited a total of 1,956 citations, of which 70 were removed for being duplicates, leaving 1,889 unique studies (see table 1 and figure 1– PRISMA flow diagram [40] below).

**Table 1:** Database search results.

DETAILS	DATABASES					TOTAL
	PUB MED	SCOPUS	COCHRANE	CINAHL	PSYCH INFO	
Records identified through database searching	842	999	8	9	98	1956
Records selected after title screening	90	157	0	0	13	260

Following title and abstract screening, 51 studies went through to full text assessment of eligibility. Following this phase, 39 studies were included in the assessment. Six studies (DP16, [41] DP17, [42] DP22, [43] DP23, [44] DP24, [45] and DP25 [46]) were excluded in the primary validity assessment (Table S1 in Appendix S1 – primary validity assessment and Table S2 – secondary validity assessment). The remaining 20 studies that were scored “Done” proceeded to the secondary validity assessment of methodological details. Five studies (DP4, [47] DP13, [48] DP15, [49] DP20, [50] and DP21 [51]) were excluded in the secondary validity assessment, leaving 15 studies for data analysis. In total, 11 studies were excluded during validity assessment, leaving 15 studies for data analysis. There were no disagreements that required an arbiter.

### Baseline Characteristics

Of 15 studies included in data analysis, 13 studies (PC1, [52] DP1, [53] DP2, [54] DP3, [55] DP5, [56] DP6, [57] DP8, [58] DP9, [59] DP10, [60] DP11, [61] DP14, [62] DP18 [63] and DP19 [64]) were identified as RCTs, one CCT (DP12 [65]) and one CBA (DP7 [66]). Of these, only one trial (PC1) was included in the pre-conceptual group. It was not however possible, to assess more than one pre-conceptual study as the internal validity of most of the studies was severely compromised due to poor design and therefore they were excluded. Baseline characteristics are summarised in table 2 below:

**Table 2:** Summary of baseline characteristics.

ID	Country	Year of research	Study design	Mean age*	Mean pre-pregnancy BMI*	Gestation week at recruitment*	Parity -Nulliparous	Other health behaviour
				years	Kg/m2	weeks	(%)	
PC1	USA	N/A	RCT	26.5±5.02	N/A	N/A	N/A	N/A
DP1	USA	2006–2008	RCT	28.6±5.2	26.3±5.6	13.6±1.8	76.3	N/A
DP2	USA	2006–2007	RCT	26.5	27.3±7.0	19.1±6.0	53	N/A
DP3	Australia	2007–2008	RCT	30.3±5.3	35.2±7.0	N/A	N/A	4% smokers
DP5	Finland	2002–2005	RCT	26.5	23·6±3·8	N/A	58	18.5% smokers
DP6	Belgium	2006–2008	RCT	28.3±4.0	34.1±4.5	9.7±2.6	42.3	6.2% smokers
DP7	Sweden	2003–2005	CBA	30.0±4.7	N/A	10–12	41.9	8.4% smokers
DP8	USA	1998–2005	RCT	26.8	N/A	12 to 28	16.3	N/A
DP9	Australia	2007–2008	RCT	N/A	N/A	14	N/A	N/A
DP10	USA	2005–2007	RCT	26.7±6.0	25.6±6.0	13.6±3.6	45.6	5.4% smokers
DP11	Denmark	N/A	RCT	28±4.0	34.9±4	15±2	N/A	N/A
DP12	Finland	2004–2005	CCT	27.6±4.5	23.7±3.9	8–9	100	32% smokers
DP14	USA	N/A	RCT	25.5±4.8	26.9±4.4	14.5±3.5	47	N/A
DP18	Canada	2004–2005	RCT	26.2±5.4	23.4±3.9	<26	N/A	N/A
DP19	Norway	2007–2008	RCT	31.2±3.7	23.8±3.8	17.3±4.1	100	4% smokers

*Mean ± Standard Deviation; N/A  =  Not applicable or Not reported; CBA  =  Control before and after; RCT  =  Randomised controlled trial; CCT  =  Controlled clinical trial.

### Types of Participants

In total, 3,426 participants were enrolled in 15 studies included in the review (table 3). Of these, 692 participants were enrolled in the pre-conceptual group and 2,734 participants in the pregnancy group. In total, 2,669 participants completed the first follow-up assessment, of which 362 belonged to pre-conceptual group and 2,307 belonged to pregnancy group. The first follow-up assessment of the pre-conceptual group was conducted 14 weeks after the intervention and in the pregnancy group it ranged from 12-week gestation to 12 month postpartum (24 month for infant only). In total, 694 (20.2%) participants were lost during the first follow-up. Of these, 330 (47.6%) belonged to the pre-conceptual group and 364 (13.3%) belonged to the pregnancy group. Each follow-up point demonstrated the trend in progressive loss to follow-up with time (table 3):

**Table 3:** Summary of participants.

ID	Participants included		Assessment of participants	Participants included in the study [N]	First follow-up		Last follow-up	
	Type	Classification			Number completing [n]	Loss to follow up [n (%)]	Number completin g [n]	Loss to follow up [n (%)]
PC1	UW, NW, OW, OB	All weight	At entry, 14 weeks, 6 and 12 months, birth records	692	362	330 (47.6%)	302	390 (56.3%)
DP1	NW, OW, OB	Normal weight, overweight and obese	At entry, 30 week gestation, 6 month postpartum	401	363	38 (9.4%)	358	43 (10.7%)
DP2	UW, NW, OW, OB	All weight	At entry and 6.1 weeks after the intervention	321	287	34 (10.5%)	N/A	N/A
DP3	OB	Obese	At entry, 12, 20 28 and 36 weeks gestation and 6 weeks postpartum	50	40	10 (20.0%)	36	14 (28%)
DP5	UW, NW, OW	Underweight, normal weight and overweight	At entry, 6, 12 months postpartum and 24 month infant only.	256	185	27 (10.5%)	191	65 (25.3%)
DP6	OW, OB	Overweight and obese	At entry (first trimester), second and third trimester, at delivery	176	122	54 (30.6%)	N/A	N/A
DP7	OB	Obese	At entry, at delivery	368	348	20 (5.4%)	N/A	N/A
DP8	OB	Obese	At entry, 6 week postpartum	257	232	25 (9.7%)	N/A	N/A
DP9	UW, NW, OW, OB	All weight	At entry and 36 weeks gestation	286	236	50 (17.4%)	N/A	N/A
DP10	NW, OW, OB	Normal weight, overweight and obese	At entry and 3–6 month postpartum	144	100	38 (26.3%)	N/A	N/A
DP11	OB	Obese	At entry, 27 and 36 week gestation, at delivery	66	50	16 (24.2%)	35	15 (22.7%)
DP12	NW, OW	Normal weight and overweight	At entry and 36–37 weeks°gestation.	132	105	27 (20.0%)	N/A	N/A
DP14	NW, OW, OB	Normal weight, overweight and obese	At entry, 30 weeks gestation, 6 weeks postpartum	120	110	10 (8.3%)	74	46 (38.3%)
DP18	OB	Obese	At entry, postpartum	52	45	7 (13.5%)	N/A	N/A
DP19	UW, NW, OW, OB	All weight	At entry, 36–38 week gestation and 6–12 weeks after delivery (postpartum).	105	84	8 (7.6%)	90	10 (9.52%)

N/A  =  Not applicable or Not reported; UW  =  underweight; NW  =  normal weight; OW  =  overweight; OB  =  obese.

### Types of Interventions

In total, nine studies primarily focused on the prevention of obesity (PC1, DP1, DP2, DP3, DP5, DP6, DP9, DP10 and DP12) and the remaining six studies focused on the treatment of obesity (DP7, DP8, DP11, DP14, DP18 and DP19). In two studies, two different interventions were compared to standard maternity care (DP6) or placebo/no interventions (DP5). These studies were further sub-divided into two sub-studies (DP6-Passive and DP6-Active; DP5-1 and DP5-2). Therefore, of 15 studies, 17 lifestyle interventions in total were identified (table 4).

**Table 4:** Summary of interventions.

ID	Intervention	Content of intervention				Delivery of intervention	Setting	Beginning of intervention
	Type	Diet	Physical activity	Motivational talks	Feedback and/or weight monitoring			Trimester*
PC1	Passive	Yes	Yes			Healthcare professionals	Clinic	Preconception; Inter-conception
DP1	Passive	Yes	Yes	Yes	Yes	Healthcare professionals	Clinic	Second
DP2	Passive	Yes	Yes	Yes		N/A or self administered	Clinic	Second
DP3	Passive	Yes	Yes	Yes		Healthcare professionals	Clinic	Not stated
DP5–1	Pro-active	Yes				Healthcare professionals	Clinic	Not stated
DP5–2	Pro-active	Yes				Healthcare professionals	Clinic	Not stated
DP6– Passive	Passive	Yes	Yes			N/A or self administered	Clinic	First and/or second
DP6– Active	Passive	Yes	Yes			Healthcare professionals	Clinic	First and/or second
DP7	Pro-active	Yes	Yes	Yes		Healthcare professionals	Clinic	Second
DP8	Passive	Yes	Yes		Yes	Healthcare professionals	Clinic	Second and/or third
DP9	Passive				Yes	Healthcare professionals	Clinic	First and/or second
DP10	Passive	Yes	Yes	Yes	Yes	Healthcare professionals	Clinic	Second
DP11	Passive	Yes			Yes	Healthcare professionals	Clinic	Second
DP12	Pro-active	Yes	Yes	Yes		Healthcare professionals	Clinic	First
DP14	Passive	Yes	Yes	Yes	Yes	Healthcare professionals.	Clinic	Second
DP18	Pro-active	Yes	Yes		Yes	N/A or self administered	Community	Third
DP19	Pro-active		Yes			Certified aerobics instructor	Community	Second

*Trimester: up to 12 =  first, 12–20 =  second, more than 20 =  third.

**Figure 2:**
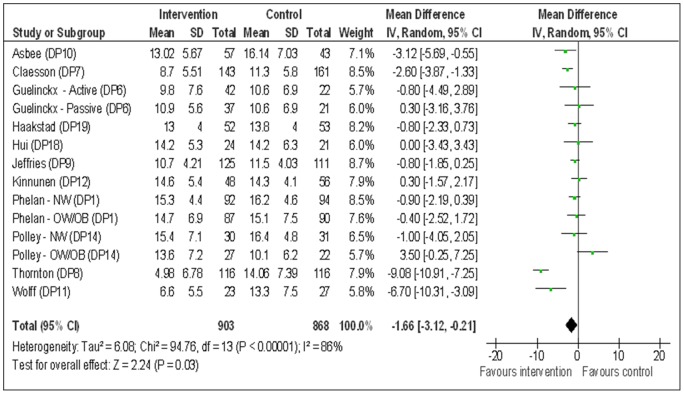
Forest plot comparing gestational weight gain (kg) between those with behaviour change interventions during pregnancy and those with standard maternity care.

**Figure 3:**
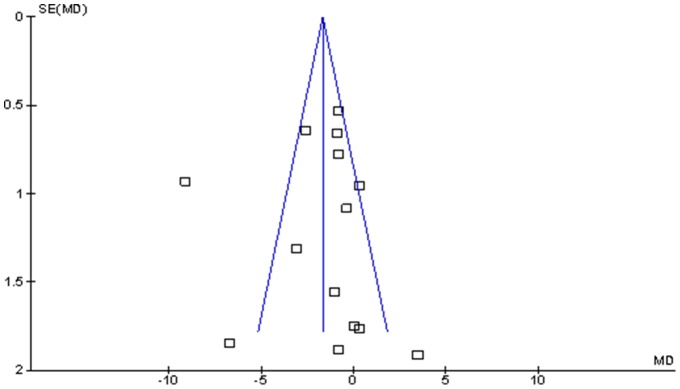
Funnel plot comparing gestational weight gain (kg) between those with behaviour change interventions during pregnancy and those with standard maternity care.

Interventions based on advice alone were called passive interventions and the interventions that provided physical activity and diet to the participants were called pro-active interventions. Of 15 studies, eight studies provided advice on diet and physical activity (PC1, DP1, DP2, DP3, DP6, DP8, DP10 and DP14) of which four studies also provided regular feedback and/or weight monitoring of the participants (DP1, DP8, DP10, and DP14) and five provided motivational talks on weight gain in pregnancy (DP1, DP2, DP3, DP10 and DP14). Only one study provided advice on diet alone (DP11) supplemented by feedback on progress. Provision of food/nutrients and physical activity was provided in three studies (DP7, DP12 and DP18) of which two studies also provided motivational talks on weight gain in pregnancy (DP7 and DP12). In two studies provision of diet or physical activity was provided (DP5 and DP19).

The beginning of interventions in the pregnancy group ranged from six to 30 weeks gestation (table 4). One study was delivered in-group (DP19) and eight were delivered individually (DP1, DP3, DP6, DP8, DP9, DP10, DP11 and DP14). Five studies were delivered in a group as well as individual (PC1, DP5, DP7, DP12 DP18). One study (DP2) had no clear statement on the delivery of the intervention to the participants. Nine studies were delivered face to face (DP3, DP5, DP6, DP7, DP8, DP10, DP11, DP12 and DP19), one was delivered as a video (DP2), three studies were delivered remotely by phone and face to face (PC1, DP1 and DP14) and one was delivered face to face and by computer (DP18). Mode of delivery was unclear in one study (DP9).

### Outcomes of Interest

Only two studies (PC1 and DP19) used the current 2009 IOM pregnancy guidelines and one study used the Canadian guidelines on healthy weights (DP18) when stating GWG (Table S3 in Appendix S1 - reported outcomes of interest). The rest of the 12 studies used 1999 IOM pregnancy guidelines. All studies reported on GWG except for one study (DP3). Three studies reported on postpartum weight loss (DP1, DP8 and DP14). Four studies reported on postpartum weight retention (DP1, DP7, DP14 and DP19). Eight studies reported on both infant birth weight and gestational week of delivery (DP1, DP5, DP6, DP7, DP8, DP9, DP14 and DP18) and two studies reported on infant birth weight (DP12) or gestational week of delivery alone (DP11).

The pre-conceptual group (PC1) reported significantly less GWG in the intervention group (P = 0.023). The impact of intervention on postpartum weight loss and retention, infant birth weight and gestational week of delivery was not reported in the pre-conceptual group.

Four studies in the pregnancy group reported significantly less GWG in the intervention group (DP7, DP8, DP10 and DP11). While two studies showed significantly less GWG in normal weight women only (DP1, and DP14), one study reported significantly less GWG in overweight participants (MD 0.12 kg, 95% CI 0.03 to −0.22, P = 0.01) belonging to the intervention group (DP9).

### Risk of Bias in Included Studies

A rigorous quality assessment of the studies was carried out (table 5). The quality assessment scores ranged from 4–12, with higher scores indicating greater quality. Figure S4 in Appendix S1 – Judgements about each risk of bias and Figure S2 in Appendix S1 – Risk of bias graph.

**Table 5:** Summary of risk of bias.

ID	SELECTION BIAS		PERFORMANCE BIAS	ATTRITION BIAS		REPORTING BIAS	TOTAL QUALITY SCORE
	Random sequence generation	Allocation concealment	Blinding of outcome assessor and analyst	Incomplete outcome data	Intention to treat analysis	Selective outcome reporting	
PC1	2	1	0	0	2	0	5
DP1	2	2	2	2	2	2	12
DP2	2	1	0	2	2	0	7
DP3	2	0	1	2	0	0	5
DP5	2	1	2	2	2	1	10
DP6	1	1	0	0	0	2	4
DP7	0	N/A	1	2	0	2	5
DP8	2	2	0	2	2	2	10
DP9	2	2	0	2	2	0	8
DP10	2	2	0	0	0	0	4
DP11	2	1	2	0	0	0	5
DP12	0	N/A	0	2	0	2	4
DP14	0	1	1	2	2	2	8
DP18	1	1	1	2	0	2	7
DP19	2	1	2	2	2	2	11

N/A  =  Not applicable; 0 =  Inadequate 1 =  Unclear; 2 =  Adequate.

## Meta-Analysis – Result

### Effects of Interventions

#### Effects of behaviour change interventions in pregnancy versus standard maternity care

The pregnant women in the intervention groups showed statistically significant reduction in GWG compared to standard maternity care groups [14 study groups, n = 1771, mean difference (MD) −1.66 kg, 95% confidence interval (CI) −3.12 to −0.21], random-effects analysis.

There was no statistically significant difference identified between the interventions and standard maternity care groups for:

- Postpartum weight loss [five study groups, n = 669, mean difference (MD) 0.21 kg, 95% confidence interval (CI) −0.58 to 1.01], fixed-effects analysis;
- Postpartum weight retention [six study groups, n = 839, mean difference (MD) −0.99 kg, 95% confidence interval (CI) −2.25 to 0.26], random-effects analysis;
- Gestation week of delivery [eight study groups, n = 1146, mean difference (MD) 0.19 week, 95% confidence interval (CI) −0.03 to 0.41], fixed-effects analysis.

In addition, the infants of pregnant women assigned to behavioural interventions showed no statistically significant difference in the birth weight compared to infants of pregnant women assigned to standard maternity care [nine study groups, n = 1381, mean difference (MD) 17.88g, 95% confidence interval (CI) −38.93 to 74.69], fixed-effects analysis. The summary of meta-analysis on outcomes of interest is shown in Table 6.

**Table 6:** Summary of data analysis on outcomes of interest.

Outcome	Study groups	Participants	Statistical method	Effect estimate
Gestational weight gain	14	1771	Mean Difference (Random, 95% CI)	−1.66 [−3.12, −0.21]
Postpartum weight loss	5	669	Mean Difference (Fixed, 95% CI)	0.21 [−0.58, 1.01]
Postpartum weight retention	6	839	Mean Difference (Random, 95% CI)	−0.99 [−2.25, 0.26]
Gestation week of delivery	8	1146	Mean Difference (Fixed, 95% CI)	0.19 [−0.03, 0.41]
Infant birth weight	9	1381	Mean Difference (Fixed, 95% CI)	17.88 [−38.93, 74.69]

### Gestational weight gain

There was considerable heterogeneity between studies in GWG group, as indicated by Higgins I^2^ [Tau^2^ = 6.08; Chi^2^ = 94.76, df = 13 (P<0.00001); I^2^ = 86%]. Higgins I^2^ also denoted substantial heterogeneity between studies in postpartum weight retention group [Tau^2^ = 1.42; Chi^2^ = 14.05, df = 5 (P = 0.02); I^2^ = 64%]. However, no heterogeneity was observed between studies for postpartum weight loss, gestation week of delivery and infant birth weight. Therefore, random-effect meta-analysis was used when reporting GWG and postpartum weight retention and fixed-effect meta-analysis was used when reporting the other three outcomes. The analysis of each study in the forest plot is described below for each outcome.

Of 14 study groups, only four study groups (DP7, DP8, DP10 and DP11) generated significant effect with the intervention groups gaining 2.60 to 9.08 kg less than the control groups. Ten study groups did not show any evidence of effectiveness between the groups (DP1-NW, DP1-OW/OB, DP6-Active, DP6-Passive, DP9, DP12, DP14-NW DP14-OW/OB, DP18 and DP19) (Figure: 2). The study by Thornton (DP8) showed by far the strongest effect estimate of all included studies, with the pregnant women in the intervention group gaining a mean 9.08 kg, (95% CI −10.91 to −7.25) less than the women in control group during pregnancy. The study by Wolff (DP11) also showed a strong effect estimate although it was smaller with 50 participants compared to 232 in Thornton's. Both studies enrolled obese participants only, which may help to explain their results. The funnel plot was fairly symmetrical for GWG indicating non-evident publication bias (Figure: 3).

### Subgroup Analysis

#### Effects of behaviour change interventions in pregnancy versus standard maternity care: Gestational weight gain outcome variations

A significant difference in the percentage of Higgins I2 in each of the subgroups was observed during the analysis. The summary of subgroup analysis of GWG outcomes is shown in Table 7.

**Table 7:** Summary of subgroup analysis of gestational weight gain.

Subgroup	Study groups	Participants	Statistical method	Effect estimate
Variation in types of participants				
All weight	2	341	Mean Difference (Random, 95% CI)	−0.80 [−1.67, 0.07]
Obese	4	631	Mean Difference (Random, 95% CI)	−4.65 [−8.74, −0.56]
Variation in practice setting				
Hospital	12	1621	Mean Difference (Random, 95% CI)	−1.85 [−3.53, −0.18]
Community	2	150	Mean Difference (Random, 95% CI)	−0.67 [−2.06, 0.73]
Variation in quality of studies				
Low quality	6	680	Mean Difference (Random, 95% CI)	−2.00 [−3.80, −0.20]
High quality	8	1091	Mean Difference (Random, 95% CI)	−1.36 [−3.55, 0.84]
Variation in type of interventions				
Passive	10	1213	Mean Difference (Random, 95% CI)	−1.98 [−4.10, 0.13]
Pro-active	4	558	Mean Difference (Random, 95% CI)	−1.00 [−2.47, 0.47]
Variation in content of intervention (based on psychological theory)				
Regular feedback and/or weight monitoring	9	1136	Mean Difference (Random, 95% CI)	−2.13 [−4.38, 0.13]
No feedback and/or weight monitoring	5	635	Mean Difference (Random, 95% CI)	−0.98 [−2.28, 0.32]

There was no significant effect of behavioural interventions targeted at all weight pregnant women on reduced GWG in the intervention groups when compared with women in the control group receiving standard maternity care (MD −0.80 kg, 95% CI −1.67 to 0.07). However, when restricted to only high quality studies, there was no significant difference (MD −1.36 kg, 95% CI −3.55, 0.84).

Interventions were more significant when delivered in the hospital setting resulting in a mean difference of −1.85 kg (95% CI −3.53 to −0.18) but were not found to be significant when delivered in the community (MD −0.67 kg, 95% CI −2.06 to 0.73). There was a significant effect of behavioural interventions targeted specifically at obese pregnant women (pre-pregnancy BMI ≥ 30 kg/m^2^) on reduced GWG in the intervention group, resulting in a mean difference of −4.65 kg (95% CI −8.74 to −0.56).

There was no significant effect of passive behavioural interventions targeted at pregnant women on reduced GWG in the intervention groups when compared with women in the control group receiving standard maternity care (MD −1.98 kg, 95% CI −4.10 to 0.13) and similarly no significant effect of pro-active behavioural interventions (MD −1.00 kg, 95% CI −2.47 to 0.47).

When analysed by the content of the intervention, there was no significant effect of behavioural interventions with feedback and/or weight monitoring of participants targeted at pregnant women on reduced GWG in the intervention groups, when compared with women in the control groups receiving standard maternity care (MD −2.13 kg (95% CI −4.38 to 0.13) and similarly no significant effect of behavioural interventions with no feedback and/or weight monitoring (MD −0.98 kg, 95% CI −2.28 to 0.32).

### Sensitivity Analysis

**Figure 4:**
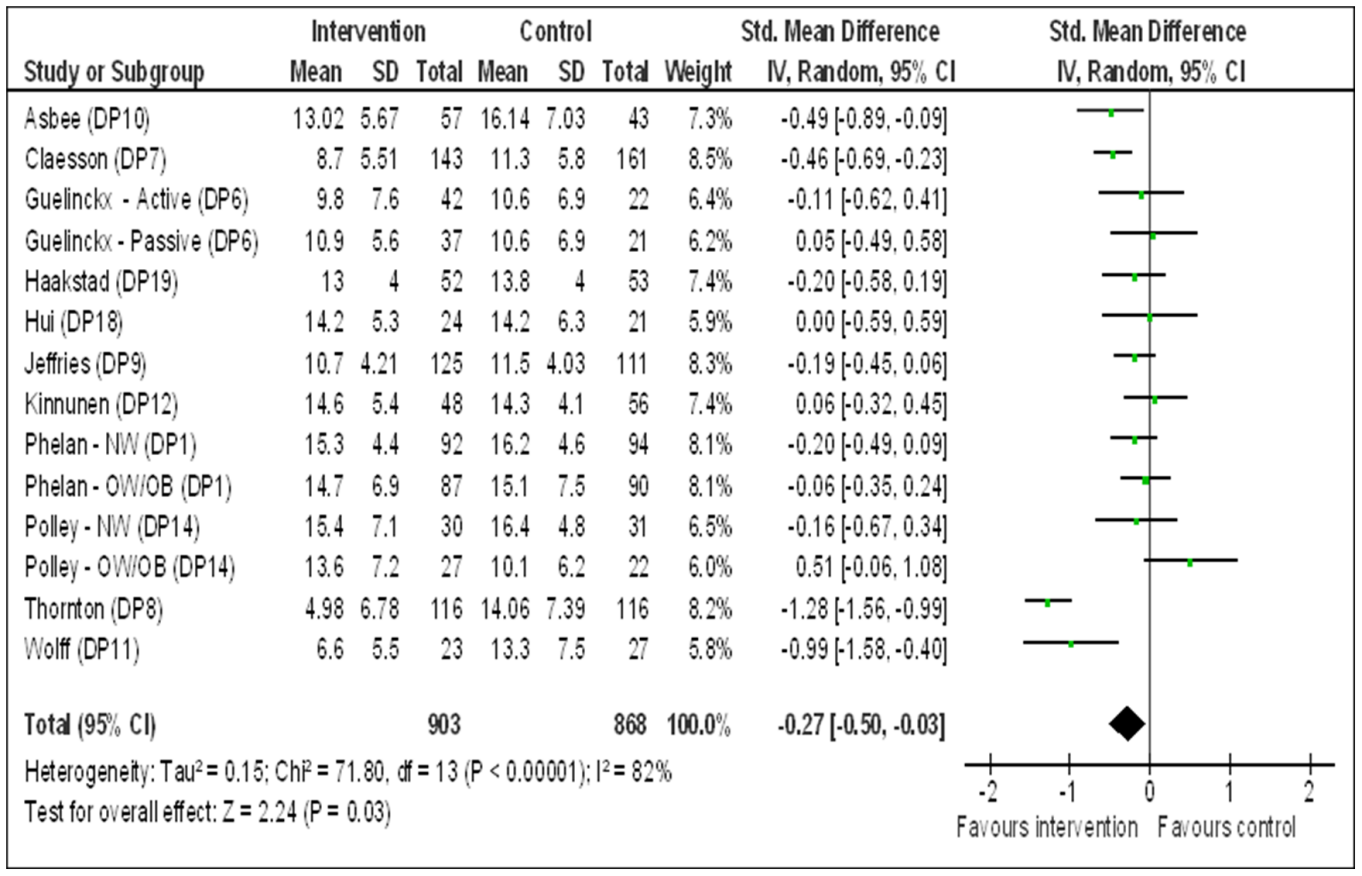
Forest plot comparing gestational weight gain (standardised mean difference in kg) between behaviour change interventions during pregnancy versus standard maternity care – sensitivity analysis.

Changing the analysis method from mean difference to standardised mean difference (SMD) across GWG outcome scales, resulted in a SMD of −0.27 kg (95% CI −0.50 to −0.03). Therefore, the forest plot across all study groups included in GWG outcome analysis indicated a reduction in GWG on average in the intervention groups when compared to standard maternity care in the control groups (Figure 4 below).

A considerable heterogeneity was observed between the study groups as indicated by Higgins I^2^ [Tau^2^ = 0.15; Chi^2^ = 71.80, df = 13 (P<0.00001); I^2^ = 82%]. However, the value for Chi^2^ and I^2^ is significantly lower in SMD scale compared to MD scale.

## Discussion

Poor weight management during pregnancy can have potentially adverse effects on mother and baby [6]–[7]. Low birth weight increases the risk of coronary heart disease, as stated in the “Barker theory” [67]–[68]. However, despite numerous articles on the prevalence and implications of maternal weight on the health of the mother and infant, few interventions for gestational weight gain have been suggested.

Several meta-analyses [69]–[72], have been published on gestational weight management interventions with an emphasis on diet and physical activity. However, none of the reviews have specifically identified the main features of effective interventions and their impact on maternal and neonatal outcomes. This review summarises 15 studies (14 in the pregnant women group and only one in the pre-conceptual group) involving 3,426 women. The systematic review results have shown that dietary and physical activity counselling at the pre-conceptual stage results in a reduction in GWG in the intervention group. However, there is currently limited information to base clinical recommendations about the efficacy of implementing interventions at this stage, since only a single study [41] was found looking at the pre-conceptual group.

Upon synthesis of the studies, the interventions produced a small but significant average reduction of 1.66 kg (95% CI 3.12 to 0.21, range – increase of 3.50 kg to reduction of 9.08 kg) in GWG in relation to the mean GWG of 12.6 kg in the 1,771 women who completed the studies. This is thought to be a clinically significant reduction [70]–[71]. However, further research is needed to confirm the health benefits and to determine their magnitude. The techniques that were most commonly used in the successful interventions involved physical activity and diet counselling by a dietician, physician or a midwife supplemented by motivational talks on weight management, feedback on the progress of participants and weight monitoring during pregnancy. The frequency of diet counselling ranged from one session (DP8) to sessions at each clinical visit (DP7). However, the most noticeable difference of 9.08 kg reduction in the GWG of intervention group was observed in the study where diet and physical activity counselling (supplemented by weight monitoring at each clinical visit) was provided to obese pregnant women (DP8).

In interventions where diet and physical activity counselling were not used together, intense diet counselling for up to 10 times for one hour during pregnancy (DP11), was used to increase the intensity of intervention. Therefore, for effective weight management in pregnancy intensely delivered interventions could be promising. The interventions delivered in early pregnancy on average showed better results than those delivered later in pregnancy.

Sensitivity analysis made very little difference to the overall effect estimates. The subgroup analysis of the GWG study groups yielded that women's pre-pregnancy weight, mode of delivery of the intervention and the quality of study appeared to influence the effectiveness of the intervention. The analysis based on the psychological contents of the intervention did not demonstrate any difference in the effect of intervention between the two groups. However, in the current NICE guidelines recommendations were made against repeated weight measurements in pregnancy (unless required) [73]. When the analysis was confined to high quality studies only, there was no trend of lower GWG in the intervention groups. The analysis showed that weight management interventions are more likely to impact GWG in high-risk participants. Simultaneously, it would also appear likely that the observed effects are due to the mode of delivery of the intervention rather than the intervention itself. This shows that when interventions are based on clinical evidence and delivered by healthcare professionals, it increases the reliability of well-being for both mother and foetus [74]. However, the number and size of studies delivered in the community settings were less compared to the hospital settings. Therefore, caution is needed in their interpretation.

The observed effect on GWG and postpartum weight retention showed strong heterogeneity and was unlikely to be explained by publication bias, as indicated by a symmetrical funnel plot. There was no evidence of interventions targeting GWG to impact postpartum weight loss, postpartum weight retention, gestation week of delivery and infant birth weight. There was no data on whether babies were small for gestational age.

### Overall Completeness and Applicability of Evidence

Since it was not possible to blind the participants to the interventions, it is likely that the delivery of interventions might have generated “Hawthorne-effects,” which might have motivated the intervention group to adhere to the regime and strive to create a successful outcome. But the outcome of the intervention may not be significant when applied to real life. Therefore, to achieve more valid results, participants and assessors should be randomised and blinded to the interventions.

In two studies (DP8 and DP11) only obese participants were included and their GWG was measured by last weight before delivery minus self-reported pre-pregnancy weight. Obese participants may have overestimated their pre-pregnancy weight with the corollary of a lower GWG. On average, during pregnancy seven to eight pounds (3.18–3.63 kg) of extra weight is contributed by the weight of the baby and the rest by placenta, amniotic fluid, uterus, maternal blood, fluids in maternal tissue and maternal fat and nutrient stores [75]. In these participants, the mean GWG in the intervention group was 4.98 kg (DP8) and 6.6 kg (DP11) and mean infant birth weight was 3.52 kg (DP8) and 3.75kg (DP11). One should also bear in mind how diurnal variation in participant hydration could potentially represent a confounder in weight measurements, although it should affect groups equally in good RCTs. Finally, since we excluded those with comorbid conditions like diabetes and hypertension, the results apply only to those without comorbidities.

### Methodological Quality of the Evidence

All the studies were conducted in high income countries only, which may not be generalisable to low and middle income countries due to lack of resources and expertise. None of the studies were conducted in the UK. The data collection and reports on findings of studies were conducted in different cultural and healthcare system settings (non-NHS) and may not be generalisable to the UK, which is a limitation of this research. Some studies were of poor quality and generally small with insufficient numbers of patients. In many cases the published reports were inadequate for our purpose and there was also incomplete reporting of data in some studies. The majority of the studies were reporting impacts of intervention on GWG only, so enough evidence cannot be collected for the maternal and neonatal outcomes. In addition, a standardised method for measuring weight (such as pre-breakfast) was not deployed amongst all the studies. With the differential for GWG in intervention and control studies being so small, this could potentially be an important source of bias.

Only eight out of 15 studies utilised an intention to treat analysis and none mentioned crossover and its likely impact on the validity of randomisation. Considerably increased attrition or loss to follow-up (≥20%) was noticed in included studies, which may have affected the quality of the evidence. The limitations of this review also stem from the methodological insufficiencies and the considerable heterogeneity in the studies and incomplete reporting of objectives due to unavailability of data. In addition out of 15 studies, only four had specifically reported adequate blinding of the outcome assessor.

Of all the studies included in the review, only two studies (PC1 and DP19) were based on current 2009 IOM pregnancy guidelines. Therefore, further studies on interventions are needed based on current IOM guidelines. Following the consensus statement by the RCM, RCOG and RCGP, interventions may include provision of training to healthcare professionals for effective delivery of interventions [76].

### Potential Biases in the Review Process

The search was not reviewed by a librarian and was limited to scientific papers in the English language from January 2000 to May 2011 only. The English language has increasingly become the lingua franca of science with an estimated 80–90% of papers in scientific journals written in English [77]. This has been coupled with initiatives to offer translation services of key papers [78]. We feel that this strategy would still capture all the relevant papers.

In the meta-analysis, non-randomized studies were also included in order to increase statistical power. However, non-randomized studies may be biased due to structural disparities between the intervention and control groups and potential hidden confounders. Also due to differences in the reporting of socio-economic status in each study, this baseline characteristic was not considered in the discussion of results.

### Agreements and Disagreements with Other Meta-Analysis

This systematic review of studies found that there is some evidence to determine whether interventions can moderate GWG in pregnant women. Compared to previous meta-analyses [69]–[72] there is a variation in the overall reported mean difference between the groups in this review. Other reviews stated no clear conclusion and noted that further research is needed [31], [79] However, this review provided an important new insight into the effects of interventions to reduce GWG by quantifying the strength of the effect. Of five meta-analyses published on dietary and/or physical activity interventions to reduce GWG in pregnant women, only three studies identified reduction in GWG [70]–[71] However, other studies provided evidence on no effect of interventions on GWG between the control and intervention groups [80]–[81].

Our analysis disagrees with the findings of one study [71] that physical activity combined with diet and weight monitoring seem to impact GWG and interventions confined to only one of the domains does not impact GWG. In our analysis interventions confined to diet only showed evidence of reduced GWG in the intervention group.

More recently, Thangaratinum et al [72] found that dietary interventions were the most effective type of intervention for reducing GWG in pregnancy, as well as gestational hypertension and shoulder dystocia. Their meta-analysis of 30 RCTs showed a reduction in GWG of 0.97 kg, compared to 1.66 kg found in our study. This may be reflective of the different RCTs incorporated into the meta-analysis. Thangaratinum et al found that dietary interventions achieved a greater reduction in GWG than physical activity or those with a mixed approach. However, our work demonstrates that further work is needed to determine the optimum regime. There is now an overriding need for RCTs adequately powered for clinical outcomes and which can delineate which aspect of the intervention yielded the greatest results. Examples like the Australian LIMIT trial [81] and the UPBEAT trial [82] in the UK are such examples. Further work on developing core standardised outcome measures like the COMET initiative may yield fruitful results [83].

## Conclusions

Our findings suggest that behavioural interventions in pregnancy when delivered to obese women without comorbid conditions may be effective in reducing GWG. However, significant heterogeneity exists amongst those studies involving obese participants. This review did not find statistically significant evidence for behavioural interventions delivered during pre-conception or pregnancy significantly impacting on postpartum weight loss, postpartum weight retention, gestation week of delivery and infant birth weight across overweight, obese and morbidly obese women. There is a lack of appropriately designed, high-quality studies on weight management in pre-conceptual women. Further work is needed to determine the optimum intervention, its intensity, timing and setting for significant reductions in GWG and to quantify the health benefits.

## Supporting Information

Appendix S1 **Combined File Containing 3 Supporting Tables and 2 Figures.** – Table S1: Primary Validity Assessment – Table S2: Secondary Validity Assessment – Table S3: Reported Outcomes of Interest – Figure S1: Judgements about each risk of bias – Figure S2: Risk of bias graph.
(DOC)

Checklist S1 **PRSMA Checklist.**
(DOCX)
