# Correction: Interventions to Reduce and Prevent Obesity in Pre-Conceptual and Pregnant Women: A Systematic Review and Meta-Analysis

**DOI:** 10.1371/journal.pone.0110601

**Published:** 2014-10-06

**Authors:** The PLOS ONE Staff

The third author’s name is spelled incorrectly. The correct name is: Jane Sandall. The correct citation is: Agha M, Agha RA, Sandall J (2014) Interventions to Reduce and Prevent Obesity in Pre-Conceptual and Pregnant Women: A Systematic Review and Meta-Analysis. PLoS ONE 9(5): e95132. doi:10.1371/journal.pone.0095132
