# Supplementary material for: Interventions to Reduce and Prevent Obesity in Pre-Conceptual and Pregnant Women: A Systematic Review and Meta-Analysis
Source: PLoS One. 2014 May 14;9(5):e95132. doi: 10.1371/journal.pone.0095132 (PMC4020754; doi:10.1371/journal.pone.0095132)
Supplement: Appendix S1 — Combined File Containing 3 Supporting Tables and 2 Figures. – Table S1: Primary Validity Assessment – Table S2: Secondary Validity Assessment – Table S3: Reported Outcomes of Interest – Figure S1: Judgements about each risk of bias – Figure S2: Risk of bias graph. (DOC) [file pone.0095132.s001.doc]

| **ID** | **AUTHOR NAMES** | **STUDY DESIGNS** | | | | **ACTION** |
| --- | --- | --- | --- | --- | --- | --- |
|  |  | Randomised Controlled Trial | Controlled Clinical Trial | Controlled Before and After study | Interrupted Time Series |  |
| PC1 | Weisman et al,  Downs et al,  Hillemeier et al | Done |  |  |  | Include |
| DP1 | Phelan et al | Done |  |  |  | Include |
| DP2 | Jackson et al | Done |  |  |  | Include |
| DP3 | Foxcroft et al,  De Jersey et al,  Callaway et al | Done |  |  |  | Include |
| DP4 | Rhodes et al | Done |  |  |  | Include |
| DP5 | Ilmonen et al,  Luto et al,  Laitinen et al,  Aaltonen et al | Done |  |  |  | Include |
| DP6 | Guelinckx et al | Done |  |  |  | Include |
| DP7 | Claesson et al |  |  | Done |  | Include |
| DP8 | Thornton et al | Done |  |  |  | Include |
| DP9 | Jeffries et al | Done |  |  |  | Include |
| DP10 | Asbee et al | Done |  |  |  | Include |
| DP11 | Wolff et al | Done |  |  |  | Include |
| DP12 | Aittasalo et al,  Kinnunen et al |  | Done |  |  | Include |
| DP13 | Santos et al | Done |  |  |  | Include |
| DP14 | Polley et al | Done |  |  |  | Include |
| DP15 | Bechtel-Blackwell et al |  | Done |  |  | Include |
| DP16 | Gray Donald et al |  |  |  |  | Exclude* |
| DP17 | Shirazian et al |  |  |  |  | Exclude** |
| DP18 | Hui et al | Done |  |  |  | Include |
| DP19 | Haakstad et al | Done |  |  |  | Include |
| DP20 | Lombard et al | Done |  |  |  | Include |
| DP21 | Colley et al |  |  | Done |  | Include |
| DP22 | Mottola et al |  |  |  |  | Exclude** |
| DP23 | Lindholm et al |  |  |  |  | Exclude**** |
| DP24 | Olson et al |  |  |  |  | Exclude** |
| DP 25 | Piirainen et al |  |  |  |  | Exclude*** |

**Table S1: Primary Validity Assessment**

*Non-randomised historical control group; **Historical cohort; ***Prospective cohort; ****Short report

**Table S2: Secondary Validity Assessment**

| **ID** | **AUTHOR NAMES** | **METHODOLOGICAL INCLUSION CRITERIA** | | **ACTION** |
| --- | --- | --- | --- | --- |
|  |  | The quantitative measurement of health and patient outcomes | Relevant and interpretable data presented/obtainable |  |
| PC1 | Weisman et al,  Downs et al,  Hillemeier et al | Done | Done | Include |
| DP1 | Phelan et al | Done | Done | Include |
| DP2 | Jackson et al | Done | Done | Include |
| DP3 | Foxcroft et al,  De Jersey et al,  Callaway et al | Done | Done | Not clear |
| DP4 | Rhodes et al | Done | Not done | Exclude*** |
| DP5 | Ilmonen et al,  Luto et al,  Laitinen et al,  Aaltonen et al | Done | Done | Include |
| DP6 | Guelinckx et al | Done | Done | Include |
| DP7 | Claesson et al | Done | Done | Include |
| DP8 | Thornton et al | Done | Done | Include |
| DP9 | Jeffries et al | Done | Done | Include |
| DP10 | Asbee et al | Done | Done | Include |
| DP11 | Wolff et al | Done | Done | Include |
| DP12 | Aittasalo et al,  Kinnunen et al | Done | Done | Include |
| DP13 | Santos et al | Not done | Not done | Exclude** |
| DP14 | Polley et al | Done | Done | Include |
| DP15 | Bechtel-Blackwell et al | Not done | Not done | Exclude** |
| DP18 | Hui et al | Done | Done | Include |
| DP19 | Haakstad et al | Done | Done | Include |
| DP20 | Lombard et al | Not done | Not done | Exclude* |
| DP21 | Colley et al | Not done | Not done | Exclude** |

* Intervention and Population does not pass the inclusion criteria; **No quantitative assessment was made of the intervention; *** Intervention does not pass the inclusion criteria.

**Table S3: Reported Outcomes of Interest**

| **REPORTED OUTCOMES OF INTEREST** | | | | | |
| --- | --- | --- | --- | --- | --- |
| **ID** | **GWG** | **Postpartum weight loss** | **Postpartum weight retention** | **Infant birth weight** | **Gestation week at delivery** |
| PC1 | Yes | No | No | No | No |
| DP1 | Yes | Yes | Yes | Yes | Yes |
| DP2 | Yes | No | No | No | No |
| DP3 | No | No | No | No | No |
| DP5 | Yes | No | No | Yes | Yes |
| DP6 | Yes | No | No | Yes | Yes |
| DP7 | Yes | No | Yes | Yes | Yes |
| DP8 | Yes | Yes | No | Yes | Yes |
| DP9 | Yes | No | No | Yes | Yes |
| DP10 | Yes | No | No | No | No |
| DP11 | Yes | No | No | No | Yes |
| DP12 | Yes | No | No | Yes | No |
| DP14 | Yes | Yes | Yes | Yes | Yes |
| DP18 | Yes | No | No | Yes | Yes |
| DP19 | Yes | No | Yes | No | No |

Yes = Outcome reported; No = Outcome not reported

**Figure S1: Judgements about each risk of bias**

**Review authors' judgements about each risk of bias item for each included study**

Red indicates inadequate risk

Yellow indicates unclear risk

Green indicates adequate risk

**Figure S2: Risk of bias graph**

**Risk of bias graph showing review authors' judgements about each risk of bias item presented as percentages across all included studies**

Red indicates inadequate risk

Yellow indicates unclear risk

Green indicates adequate risk
